# The effect of community-based health education programs on health literacy in severely impoverished counties in Southwestern China: Results from a quasi-experimental design

**DOI:** 10.3389/fpubh.2022.1088934

**Published:** 2023-01-10

**Authors:** Benyan Li, Yuan Huang, Caitlyn Ling, Feng Jiao, Hongyun Fu, Rui Deng

**Affiliations:** ^1^School of Public Health, Kunming Medical University, Kunming, China; ^2^Department of Health Insurance, The Second Affiliated Hospital of Kunming Medical University, Kunming, China; ^3^School of Medicine, Eastern Virginia Medical School, Norfolk, VA, United States; ^4^Department of Pediatrics, Eastern Virginia Medical School, Norfolk, VA, United States

**Keywords:** health education, health literacy, poverty alleviation, quasi-experimental study, Southwestern China

## Abstract

**Background:**

A national health education program in impoverished counties to promote health literacy among rural populations was released by the Chinese government in 2018. Under this nationwide campaign, an integrated health education program was implemented in Yunnan province, which included additional culturally sensitive educational components for the severely impoverished prefectures.

**Objective:**

This study examined the differential effects of the health education program models on health literacy outcomes among residents in poverty-stricken areas.

**Methods:**

A quasi-experimental design was applied with two arms that included surveys at baseline (in October 2019) and endline (in June 2021) to collect a range of individual-level health information, including the Chinese Resident Health Literacy Scale. The intervention group received the national health education program with the additional Yunnan specific program; the control group received only the national program. Respondents were recruited *via* a multi-stage stratified sampling, including 641 participants at baseline (261 from the intervention sites and 380 from the control sites) and 693 participants at endline (288 from the intervention sites and 405 from the control sites). Chi-square and logistic regression analyses were performed to examine the association between program intervention and health literacy outcomes.

**Results:**

The overall health literacy levels were low (1.87%) at baseline, and there was no statistically significant difference between two groups (1.92 vs. 1.84%, *P* = 1.000). A significant increase (from 1.87 to 11.11%, *P* < 0.001) in the health literacy level was observed at endline in both groups. The magnitude of increase was significantly greater in the intervention group relative to the control group (17.71 vs. 6.42%, *P* < 0.001). Adjusting for the confounding factors of individual and household characteristics, results from multivariate logistic regression revealed that the odds of having adequate health literacy among participants who received both the National Program and the Yunnan Program were 3.92 times higher than those who only received the National Program (95% CI: 2.10–7.33).

**Conclusion:**

The findings highlighted the importance of incorporating non-verbal visual aids and culturally-sensitive media tools in health literacy education to address healthy lifestyle and the living contexts of the populations in poverty-stricken areas.

## 1. Introduction

Health literacy, as defined by the World Health Organization (WHO), indicates a set of knowledge, cognition, and skills that determine the motivation and ability of individuals to function effectively in maintaining and promoting health (1). A growing body of evidence has demonstrated that low health literacy is associated with poorer overall health status and higher mortality (2). People with higher levels of health literacy are more likely to use preventive health care (3) or alternative therapies (4). They are more capable of applying their knowledge and skills into health practices, thereby reducing risk of disease (5). Consequently, improving health literacy is perceived as an effective approach for raising health awareness, promoting healthy lifestyles, and improving health outcomes. Enhancing health literacy in populations through evidence-based education and lifelong learning empowers target populations and improves health equity (6). Furthermore, improving health literacy in under-resourced populations particularly contributes to alleviating poverty in developing countries (7, 8).

In China, initial interest in health literacy arose from the *Health Education and Health Promotion Guidelines (2005–2010)* issued in 2005 by the National Health Commission (9). These guidelines officially defined health literacy for the first time in China as having knowledge, skills and behaviors for personal health maintenance (9). Afterwards, the National Health Commission proposed the outline of “Chinese Resident Health Literacy-Basic Knowledge and Skills” in 2008 (10), and a nationwide survey entitled *National Heath Literacy Surveillance* (NHLS) was subsequently conducted (11) to assess the health literacy level of Chinese residents annually. In this survey, adequate health literacy was defined as correct answers to at least 80% of health literacy scale items. According to the results from the previous NHLS survey rounds, the health literacy rate of Chinese residents has increased steadily from 6.48% in 2008 to 14.18% in 2017 as a result of the nationwide health education campaigns (12). However, a significant rural-urban disparity and regional gaps in health literacy persists. Findings from the 2017 NHLS survey demonstrated a much higher health literacy rate among urban residents (19.22%) than among rural residents (10.64%). Furthermore, the health literacy rate among residents in the eastern region was higher (18.71%) than the rate (9.88%) in the western region (12). Other studies revealed that residents living in rural areas and those with low-income levels experienced severe challenges related to low health literacy, such as difficulty accessing health information and services (13–15).

In recent decades, a growing size of literature has focused on health literacy among populations worldwide (16, 17) and effective interventions to improve health literacy (18). Overall, the existing evidence supports the view that health literacy is a knowledge and skill-based capacity which can be improved through health education interventions (19) in both community-based and clinical settings (20). The effectiveness of health literacy interventions varied by locales, program design and settings in which they were implemented (21–23). While some health education programs adopted a single strategy, such as group-based intervention (24, 25) and individual contact (26, 27), other programs pursued an integrated approach to combine multiple medias and learning methods to improve efficiency and overall impact (28). A systematic review covering 27 randomized controlled trials (RCTs), 2 cluster RCTs, and 13 quasi-experimental designs on health literacy interventions revealed that programs using mixed intervention approaches had moderate effectiveness in improving knowledge, self-efficacy, adherence, quality of life and health care utilization, while the strength of evidence for single-feature interventions was low or insufficient (29).

The government of China announced a major stride in its roadmap of poverty elimination in 2012 after the 18th National Congress of the Communist Party. Targeted poverty alleviation was implemented in unprecedented scale and intensity to achieve the target of eradicating extreme poverty by 2020 (30). Although remarkable achievements had been made from 2013 to 2017, about 30 million populations in rural areas remained in poverty at the end of 2017 with 36.64% of them living in poverty due to illness (31). Recognizing the potential of health promotion in breaking the link between ill-health and poverty (7), the National Health Commission and the State Council Poverty Alleviation Office of China jointly released a national health education program in impoverished counties in 2018 to promote health literacy among rural population (32).

Yunnan province is located in the Southwest of China where 25 indigenous ethnic minorities reside. By the end of 2020, the size of population in Yunnan was 48.58 million, among which 33.6% (16.21 million) were ethnic minorities (33). According to the *Outline for Development-oriented Poverty Alleviation of China's Rural Areas (2001–2010)*, 88 counties were categorized as national-level poverty-stricken counties in Yunnan, accounting for 10.5% of the 832 poverty-stricken counties in China. Among the 88 counties, over 30% (27 counties) were further identified as severely impoverished counties (34), according to the *Implementation Opinions on Supporting Poverty Alleviation Effort in Severely Impoverished Regions* issued in 2017 by the General Office of the CPC Central Committee and the General Office of the State Council, which further categorized counties with an average poverty headcount ratio of more than 18% as severely impoverished counties. As a part of the national health education program targeting impoverished counties, the Yunnan Provincial Health Commission designed and implemented a specific health education program with an additional set of culturally sensitive educational components in 2019 in Nujiang and Shangri-La, two prefectures with high concentrations of ethnic minorities and severely impoverished counties.

In this study, we examined the effectiveness of the health education intervention and the differential effect of the program models on health literacy among residents in poverty-stricken areas in Southwestern China. We hypothesized that targeted health interventions which incorporated culturally sensitive features to address the low level of educational attainment and different cultural backgrounds would be more effective, relative to the standard national program, in improving health literacy of people living in poverty.

## 2. Methods

### 2.1. Study design

We used a quasi-experimental design which included two arms (an intervention group and a control group) and two rounds of survey data collection at baseline (in October 2019) and endline (in June 2021, 6 months after the one-year interventions). In the control group, participants were selected from poverty-stricken counties in 3 provinces in Southwestern China (including Yunnan, Guizhou and Sichuan) where the standard national health education program was implemented. In the intervention group, participants were selected from Nujiang and Shangri-La prefectures in Yunnan where the national program was implemented with additional culturally sensitive health education components. Ethical approval was obtained from Ethics Committee of the Kunming Medical University's Institutional Review Board.

### 2.2. Interventions

As mentioned above, to facilitate the Three-Year Action Plan for Poverty Elimination initiated by the Chinese Government between 2018 and 2020, the National Health Commission and the State Council Poverty Alleviation Office jointly announced a national health education program (hereinafter referred to as the National Program) in impoverished rural areas in October 2018 to improve health literacy levels of the rural residents living under the poverty line (32). The proportions of rural residents with adequate health literacy were only 5.03% in Nujiang Prefecture and 6.31% in Shangri-La Prefecture in 2018. Those percentages were far behind the national average level of 17.06% in 2018 (35). The Yunnan Provincial Health Commission designed and implemented an additional set of culturally sensitive health education components (hereinafter referred to as the Yunnan Program) in these two prefectures during 2019–2020 to improve health literacy and reduce illness-related poverty (36).

The differences between the National Program and the Yunnan Program are presented in Table 1, using Lasswell's Model of Communication which includes five key components: communicator (Who?), message (Said what?), medium (In which channel?), audience (To whom?) and effect (With what effect?) (37, 38). In the National Program, the health education team was composed mainly of trained village leaders, and primary health workers. These professionals disseminated health information developed by the national health programs for poverty alleviation at villages, households, and schools. These educational efforts included delivering lectures, pamphlets, training sessions for family members, bulletin boards, quiz competitions, public service announcements, and distribution of practical tools to promote healthy lifestyles. The specific health education materials delivered was determined by the health needs of targeted populations, which mainly included rural residents and students. Under the Targeted Poverty Alleviation Strategy, population-based household surveys at different levels were used to examine the health status and health needs of population living in poverty to inform the design of targeted health education intervention.

**Table 1:** Characteristics of two health education programs by Lasswell's model.

**Components**	**National health education program**		**Yunnan specific health education program**	
	**Strategy**	**Details**	**Strategy**	**Details**
Communicator	Trained village leaders and primary healthcare workers	Visits to villages, households, and schools visits	Health specialists from prefecture- and county-levels	Covered through lecture tours across prefectures
Message	Wide range of knowledge	• National health programs for poverty alleviation • The 66 health facts summarized by National Health Commission • Prevention and control of endemic diseases	Knowledge focused on healthy lifestyle	• Healthy diet • Weight management • Physical activity • Smoking cessation • Alcohol abstinence • Mental health • Rational use of medications • Prevention and control of chronic diseases
Medium	• A wide range of activities covering all impoverished villages, families, residents and students • Materials varied for different targeted populations	• For each village, at least one lecture every 2 months and at least one health bulletin board updated every 3 months • For each family, at least one leaflet and one practical tool distributed to promote one healthy lifestyle, and at least one family member trained • For each school in impoverished areas, health education curricula offered and quiz competitions held	• Culturally sensitive health education materials • Audio-visual materials in ethnic minority languages • Health-promotion settings	• “Healthy China” lecture covered at least 3,000 people in each county • Radio dramas in ethnic minority languages • Public-interest advertisements in ethnic minority languages
Audience	Impoverished rural residents and students	• All impoverished villages and families covered • 50% of primary and secondary schools in impoverished areas covered	• Government officials, medical doctors, and school teachers • Impoverished rural residents and students	• Government officials, medical doctors, and school teachers at county level • All impoverished villages and families covered • 50% of primary and secondary schools in impoverished areas covered
Effect	Improve health literacy levels among residents in poverty-stricken areas by 60% from 2018 to 2020		Raise the health literacy rate among local residents by 16% in 2020 in line with the provincial goal	

In contrast to the National Program, the Yunnan Program established a professional team consisting of health specialists from prefecture- and county-levels who directly conducted lecture tours across Nujiang Prefecture and Shangri-La Prefecture. A set of culturally sensitive health education materials was designed and implemented delivered to address healthy lifestyles, including healthy diet, weight management, physical activity, smoking cessation, alcohol abstinence, mental health, appropriate use of medicines, and chronic diseases prevention and control. Furthermore, audio-visual materials with the same contents were developed not only in Mandarin but also in various ethnic minority languages (e.g., Lisu, Dulong, and Zang). In addition to serving rural residents and students living in poverty, the Yunnan program also targeted the local government officials, medical doctors, and schoolteachers in the target audience to foster a friendly and supportive educational environment.

### 2.3. Participants and sampling

Baseline and endline household health literacy surveys were administered in the study sites through face-to-face interviews with trained researchers. Eligible participants were rural residents aged 15–69 years old who had stayed in the project sites for more than 6 months before the baseline survey. Those who had severe mental illness and intellectual disabilities were excluded. We conducted power analysis to estimate the sample size required to capture the anticipated effect size. The result indicated that a sample size of 500 participants, 250 in each arm, was sufficient to detect a difference of 6% between groups in health literacy score using a two-tailed *z*-test of proportions between two groups with 80% power and a 5% level of significance.

A multi-stage stratified sampling was adopted to select survey participants consistently for the intervention and control groups at the program sties. Firstly, three prefectures from Yunnan, Guizhou and Sichuan provinces in Southwestern China which shared similar geographical conditions and socioeconomic characteristics to Nujiang and Shangri-La (the intervention group) were selected for the control group Secondly, one severely impoverished county was randomly selected from each of the five prefectures by using a random number table. Then, two townships from each county, two villages from each township, and 35 households from each village were randomly selected. Thirdly, the Kish Grid was used to randomly choose an eligible participant for the survey.

### 2.4. Data collections

Data collection was implemented by post-graduate students who were trained as the survey interviewers. Baseline data were collected in October 2019 in the selected villages and households *via* a questionnaire administered face-to-face by the interviewers. Each interview took ~40-min. A total of 746 residents were invited, and 641 completed the survey, including 261 in the intervention group and 380 in the control group. The response rate of the 2019 baseline survey was 85.92%. About 6 months after the 1-year interventions, an endline survey was conducted in June 2021 at the selected program sites following the same sampling procedure. A total of 796 participants were invited and 693 completed the survey, including 288 individuals from the intervention group and 405 individuals from the control group. The survey response rate was 87.06% in 2021. All participants were fully informed about this study and gave written consent. To ensure confidentiality, data collected in this project were saved in password-locked computers and only accessible to the research team.

### 2.5. Measurements

The Chinese Resident Health Literacy Scale developed by the Chinese Ministry of Health in 2012 was adopted for both the pre- and post-intervention surveys (39). The scale has been assessed in previous studies and was proven to be valid and reliable in the contemporary social contexts in China (39). It included 50 items, which consisted of three dimensions [knowledge and attitudes (23 items), behavior and lifestyle (15 items), and health-related skills (12 items)] and six aspects [scientific views of health (8 items), infectious diseases (6 items), chronic diseases (9 items), safety and first aid (10 items), medical care (11 items), and health information (6 items)]. There were four types of questions: true-or-false, single-choice, multiple-choice and situation questions. A summary health literacy measure was calculated. Each correct answer of true-or-false, single-choice, or situation questions received 1 point; each correct answer for multiple-choice questions received 2 points. The final score ranged from 0 to 65 Adequate health literacy was defined as having correct answers to at least 80% of items across all dimensions and aspects of the health literacy scale items (39).

The survey also collected information on the socioeconomic and demographic characteristics of respondents, including gender (female or male), age (15–24, 25–44 or 45–69 years old), education (primary school, middle school or high school), marital status (single, married or separated/divorced/widowed), occupation (farmer or non-farmer), self-reported chronic medical conditions (yes or no) and household net income per capita (<10,800 or ≥10,800 Chinese Yuan). The cutoff point, 10,800 Chinese Yuan, was the national average household net income per capital in 2020 (40).

### 2.6. Statistical analyses

The Pearson chi-square test and Fisher's exact test were performed to compare the differences in socioeconomic characteristics and proportions of participants with adequate health literacy between two groups before and after intervention. Multivariate logistic regression models were performed to examine the differences in health literacy outcomes at endline between individuals who participated in the standard National Program only vs. participating in the additional Yunnan Program, adjusting for confounding factors at individual-level (including sex, age, education level, marital status, occupation, chronic disease history, and living standard) and household-level (including household income, occupation and education level of family members). The results were presented as *odds ratios* (*OR*s) with 95% *confidence intervals* (*CI*s). The log-likelihood value and R-squared value were calculated to measure the goodness of fit of the regression models. All statistical analyses were performed by using Stata 17.0. The significance level was at *P*-value <0.05 for all statistical tests.

## 3. Results

Table 2 presents the background socioeconomic and demographic characteristics between the two study groups at baseline and endline. At baseline, about half of the participants were male (48.05%) and half were between 45 and 69 years old (48.99%). The vast majority were married (79.72%), had less than middle school education (67.71%); and were farmers (77.07%). A significant proportion (14.66%) reported having at least one chronic disease. More than half were living under the national poverty line (57.10%). The vast majority (82.53%) had a household net income per capita below 10,800 Chinese Yuan (approximately equal to $1,563 USD). Few had a family member working in government sector (7.02%). Less than half (49.61%) had a female adult family member who received middle school education and above; and had any family member (42.28%) who received with a high school education or above. The background characteristics of the intervention and control groups were similar, except that there were a higher proportion of farmers (81.99 vs. 73.68%, *P* = 0.014) and a higher percentage of respondents who reported that they had at least one chronic disease in the intervention group, relative to the control group (19.54 vs. 11.32%, *P* = 0.004).

**Table 2:** Differences in socioeconomic and demographic profiles between intervention and control groups at baseline and endline.

**Background characteristics**	**Baseline (2019)**				**Endline (2021)**				**χ^2^ (*P*)**
	**All** ***n*** = **641 %**	**Intervention group** ***n*** = **261 %**	**Control group** ***n*** = **380 %**	χ^2^ **(*****P*****)**	**All** ***n*** = **693 %**	**Intervention group** ***n*** = **288 %**	**Control group** ***n*** = **405 %**	χ^2^ **(*****P*****)**	
**Gender**									
Male	48.05	49.81	46.84	0.545 (0.460)	46.75	44.44	48.40	1.055 (0.304)	0.225 (0.636)
Female	51.95	50.19	53.16		53.25	55.56	51.60		
**Age group**									
15–24	11.39	12.04	12.37	1.381 (0.501)	12.41	10.76	13.58	2.113 (0.348)	3.514 (0.173)
25–44	39.63	46.76	40.26		43.72	46.53	41.73		
45–69	48.99	62.04	47.37		43.87	42.71	44.69		
**Education level**									
Primary school or below	67.71	62.45	71.32	5.681 (0.058)	64.79	61.11	67.41	3.323 (0.190)	1.836 (0.399)
Middle school	22.78	26.05	20.53		23.67	25.35	22.47		
High school/technical school or above	9.52	11.49	8.16		11.54	13.54	10.12		
**Marital status**									
Single (never married)	11.86	12.26	11.58	1.504 (0.471)	10.82	13.19	9.14	11.527^**^ (0.003)	0.441 (0.802)
Married	79.72	77.78	81.05		81.10	75.35	85.19		
Separated/divorced/ widowed	8.42	9.96	7.37		8.08	11.46	5.68		
**Occupation**									
Farmer	77.07	81.99	73.68	6.043^*^ (0.014)	78.07	76.04	79.51	1.18 (0.277)	0.191 (0.662)
Non-farmer	22.93	18.01	26.32		21.93	23.96	20.49		
**Having any chronic disease**									
No	85.34	80.46	88.68	8.363^**^ (0.004)	80.52	73.96	85.19	13.525^***^ (< 0.001)	5.431^*^ (0.020)
Yes	14.66	19.54	11.32		19.48	26.04	14.81		
**Living under national poverty line**									
No	42.90	41.38	43.95	0.417 (0.519)	42.57	45.14	40.74	1.332 (0.248)	0.015 (0.902)
Yes	57.10	58.62	56.05		57.43	54.86	59.26		
**Household net income per capita (in Chinese Yuan)** ^a^									
< 10,800	82.53	81.99	82.89	0.0874 (0.768)	52.53	48.26	55.56	3.5887 (0.058)	135.444^***^ (< 0.001)
≥10,800	17.47	18.01	17.11		47.47	51.74	44.44		
**Any family member working in government sector**									
No	92.98	91.19	94.21	2.166 (0.141)	91.05	90.97	91.11	0.004 (0.950)	1.675 (0.196)
Yes	7.02	8.81	5.79		8.95	9.03	8.89		
**Any female adult with middle school education or above**									
No	50.39	50.57	50.26	0.006 (0.938)	49.06	47.22	50.37	0.667 (0.414)	0.235 (0.628)
Yes	49.61	49.43	49.74		50.94	52.78	49.63		
**The highest education level among family members**									
Primary school or below	24.80	22.99	26.05	1.335 (0.513)	25.69	21.53	28.64	4.601 (0.100)	1.751 (0.417)
Middle school	32.92	32.18	33.42		29.58	31.94	27.90		
High school/technical school or above	42.28	44.83	40.53		44.73	46.53	43.46		

a The cutoff point, 10,800 Chinese Yuan was the national average household net income per capital in 2020 (40).

Significance levels:

* *P* < 0.05,

** *P* < 0.01,

*** *P* < 0.001.

Results in Table 2 indicated no significant differences in the background characteristics between baseline and endline survey participants in terms of sex, age, education, marital status, occupation, and characteristics of family members. A higher proportion of participants reported having at least one chronic disease at endline, relative to baseline (19.48 vs. 14.66%, respectively *P* = 0.020). The proportion of participants who reported that the household net income per capita was more than 10,800 Chinese Yuan in 2021 increased from 17.47% at baseline to 47.47% at endline (*P* < 0.001).

Results presented in Table 2 also indicated that participants in intervention group and control groups at endline were similar in most of the background characteristics; except that a higher proportion of the respondents were married in the control group (85.19%) relative to the intervention group (75.35%; *P* = 0.003). A higher proportion of respondents in the intervention group reported having at least one chronic disease condition (26.04%) relative to participants in the control group (14.81%, *P* < 0.001).

Results in Table 3 indicated that the overall level of having adequate health literacy (having a score of 80% or above on the health literacy scale) was very low (1.87%) among the survey participants at baseline. There was no statistically significant difference between the intervention group and control group (1.92 vs. 1.84%, respectively, *P* = 1.000). Regarding the three dimensions of health literacy, the level was the lowest (1.40%) for health-related skills, followed by behaviors and lifestyles (5.46%) and knowledge and attitudes (9.36%). As for the six aspects of health literacy, the level was the lowest for health information (2.34%), and the highest for scientific views of health (19.50%). There was no statistically significant difference between the intervention and control groups in the levels and patterns of health literacy as measured by the three dimensions and six aspects of health literacy.

**Table 3:** Differences in health literacy levels between the intervention and control groups at baseline and endline.

**Health literacy**	**Baseline (2019)**				**Endline (2021)**				**χ^2^ (*P*)**
	**All** ***n*** = **641 %**	**Intervention group** ***n*** = **261 %**	**Control group** ***n*** = **380 %**	χ^2^**/Fisher (*****P*****)**	**All** ***n*** = **693 %**	**Intervention group** ***n*** = **288 %**	**Control group** ***n*** = **405 %**	χ^2^ **(*****P*****)**	
The overall health literacy	1.87	1.92	1.84	–(1.000)^a^	11.11	17.71	6.42	21.716^***^ (< 0.001)	45.650^***^ (< 0.001)
**Three dimensions**									
Knowledge and attitudes	9.36	10.34	8.68	0.503 (0.478)	23.52	32.64	17.04	22.776^***^ (< 0.001)	47.961^***^ (< 0.001)
Behavior and lifestyle	5.46	6.13	5.00	0.383 (0.536)	25.25	34.03	19.01	20.104^***^ (< 0.001)	98.346^***^ (< 0.001)
Health-related skills	1.40	1.53	1.32	–(1.000)^a^	5.19	7.64	3.46	5.977^**^ (0.014)	14.680^***^ (< 0.001)
**Six aspects**									
Scientific views of health	19.50	22.22	17.63	2.077 (0.150)	46.90	59.38	38.02	30.807^***^ (< 0.001)	111.810^***^ (< 0.001)
Infectious disease	2.96	3.45	2.63	0.359 (0.549)	8.51	10.07	7.41	1.531 (0.216)	18.629^***^ (< 0.001)
Chronic disease	12.48	14.18	11.32	1.159 (0.282)	29.73	41.67	21.23	33.637^***^ (< 0.001)	58.798^***^ (< 0.001)
Safety and first aid	19.19	19.54	18.95	0.035 (0.851)	53.39	63.54	46.17	20.404^***^ (< 0.001)	167.192^***^ (< 0.001)
Medical care	4.99	4.98	5.00	< 0.001 (0.991)	26.41	32.64	21.98	9.848^**^ (0.002)	112.955^***^ (< 0.001)
Health information	2.34	2.68	2.11	0.225 (0.635)	4.33	5.56	3.46	1.790 (0.181)	4.041^*^ (0.044)

a Fisher's exact test.

Significance levels:

* *P* < 0.05,

** *P* < 0.01,

*** *P* < 0.001.

Results in Table 3 indicated statistically significant increases in the overall level of having adequate health literacy from baseline to endline among participants in both the intervention control groups. While the level of having adequate health literacy increased from 1.84% at baseline to 6.42% at endline in the control group, the magnitude of increase was much larger in the intervention group (from 1.92% at baseline to 17.71% at endline, *P* < 0.001). The proportion of participants who had adequate health literacy in the intervention group was 11.29% higher than the control group (17.71 vs. 6.42%, respectively *P* < 0.001) at endline, indicating that the culturally sensitive additions in the Yunnan Program increased the effectiveness of the health literacy intervention among the indigenous population living in poverty-stricken areas in Yunnan.

Regarding the three dimensions of health literacy, the level of increase for the total sample was the highest for behavior and lifestyle (from 5.46 to 25.25%, *P* < 0.001) and the lowest for health-related skills (from 1.40 to 5.19%, *P* < 0.001). As for the six aspects of health literacy, the largest increase from baseline to end-line was the level of awareness of safety and first aid (from 19.50 to 46.90%, *P* < 0.001); followed by scientific views of health (from 19.19 to 53.39%, *P* < 0.001); and medical care (from 4.99 to 26.41%, *P* < 0.001). There were statistically significant differences in health literacy between the intervention group and control group, with the largest gaps being for scientific views of health (38.02 vs. 59.38%, respectively*, P* < 0.001), followed by chronic diseases (21.23 vs. 41.67%, respectively*, P* < 0.001); and safety and first aid (46.17 vs. 63.54%, respectively*, P* < 0.001). On the other hand, the levels of health literacy were consistently lower for health information (2.34 vs. 4.33%, respectively,) and infectious diseases (2.96 vs. 8.51% respectively) at both baseline and endline for the overall sample. There was no statistically significant difference on the levels of these two health literacy measures between the intervention and control groups.

Results from logistic regression on the effect of the health education intervention on health literacy are presented in Table 4. Results in Model 1 showed the crude effect of health education intervention without adjusting for confounding factors. Relative to participants who only received the National Program, the odds of having adequate health literacy were over 3 times higher [crude *OR* (C*OR*): 3.137; 95% CI: 1.904–5.169)] among participants who received both the National Program and the Yunnan Program which included additional culturally sensitive health education components..

**Table 4:** The effect of program intervention on health literacy among residents in severely impoverished counties in Southwest China–results from logistic analysis.

**Predictors**	**Model 1**		**Model 2**		**Model 3**	
	**Crude Odds Ratio**	**95% CI**	**Adjusted Odds Ratio**	**95% CI**	**Adjusted Odds Ratio**	**95% CI**
**Health education interventions**						
National program only (Ref.)	1		1		1	
National and Yunnan program	3.137^***^	(1.904–5.169)	3.821^***^	(2.066–7.069)	3.923^***^	(2.101–7.327)
**Gender**						
Male (Ref.)			1		1	
Female			1.321	(0.719–2.428)	1.200	(0.627–2.294)
**Age group**						
15–24 (Ref.)			1		1	
25–44			3.938^**^	(1.584–9.792)	4.189^**^	(1.655–10.600)
45–69			1.230	(0.385–3.934)	1.231	(0.371–4.089)
**Education level**						
Primary school or below (Ref.)			1		1	
Middle school			12.258^***^	(5.342–28.127)	8.810^***^	(3.578–21.696)
High school/technical school or above			31.016^***^	(12.120–79.370)	26.597^***^	(9.235–76.606)
**Marital status**						
Single (never married) (Ref.)			1		1	
Married			1.075	(0.427–2.705)	0.915	(0.353–2.369)
Separated/divorced/ widowed			1.660	(0.404–6.824)	1.450	(0.344–6.115)
**Occupation**						
Farmer (Ref.)			1		1	
Non-farmer			2.863^**^	(1.486–5.517)	2.824^**^	(1.428–5.583)
**Having any chronic disease**						
No (Ref.)			1		1	
Yes			0.574	(0.214–1.542)	0.544	(0.199–1.485)
**Living under national poverty line**						
No (Ref.)			1		1	
Yes			1.720	(0.899–3.289)	1.883	(0.966–3.670)
**Household net income per capita (in Chinese Yuan)**						
< 10,800 (Ref.)					1	
≥10,800					1.768	(0.951–3.290)
**Any family member working in government sector**						
No (Ref.)					1	
Yes					0.897	(0.358–2.245)
**Any female adult with middle school education or above**						
No (Ref.)					1	
Yes					1.500	(0.656–3.429)
**The highest education level among family members**						
Primary school or below (Ref.)					1	
Middle school					1.699	(0.473–6.100)
High school/technical school or above					1.216	(0.319–4.642)
Log likelihood	−231.017		−156.521		−153.218	
R-squared	0.044		0.353		0.366	

CI, Confidence Interval; Ref., reference group; Significance levels, ^*^P < 0.05,

** *P* < 0.01,

*** *P* < 0.001.

In Model 2, after controlling for confounding variables of individual characteristics, such as gender age, education, marital status, occupation, health condition, and economic status, the adjusted odds ratio (AOR) for the effect of the Yunnan Program increased to 3.821 (95% CI: 2.066–7.069).

In Model 3, when the confounding factors of individual socioeconomic background and household characteristics (e.g., household net income per capita, having a family member working in the government sector, and educational attainment of family members) were further accounted for into the model, the odds of having adequate health literacy among those who received the combined intervention increased to 3.923 (95% CI: 2.101–7.327), relative to those participated in the National Program only.

According to the log-likelihood value and R-squared value, Model 3 (−153.218 and 0.366) was a better fit than Model 1 (−231.017 and 0.044) and Model 2 (−156.521 and 0.353). After controlling for confounding factors, individuals receiving both the National Program and the Yunnan Program were 3.923 times more likely to have adequate health literacy than those only receiving the National Program.

Individuals who were between 25 and 44 years old (AOR: 4.189; 95% CI: 1.655–10.600), were non-farmers (AOR: 2.824; 95% CI: 1.428–5.583), received a middle school (AOR: 8.810; 95% CI: 3.578–21.696), or had a high school education and above (AOR: 26.597; 95% CI: 9.235–76.606) had higher odds of having adequate health literacy relative to their counterparts.

## 4. Conclusion and discussion

This study employed a quasi-experimental design to evaluate the effect of two health education interventions on health literacy in severely impoverished counties in Southwestern China. It compared the effectiveness of the National Program and the Yunnan Program, the latter of which included an additional set of culturally sensitive health education components. Health literacy was assessed among residents living in the intervention sites and control sites at baseline and endline, using the Chinese Resident Health Literacy Scale to measure the level of overall health literacy, three dimensions and six aspects of health literacy. Statistical analyses were conducted to examine the differences in health literacy across subgroups and its variation over time from baseline to endline to inform the feasibility and effectiveness of two community-based health education programs. Findings showed that the overall health literacy in the intervention group improved significantly from 1.92% in 2019 to 17.71% in 2021, and the participants in the intervention group were 3.923 times more likely to have adequate health literacy than those in the control group. Findings indicated that a combined implementation of the standard National Program and the Yunnan Program was more effective than the implementation of the National Program only for improving health literacy in populations living in poverty-stricken rural areas in Southwestern China.

Of particular interest for this study is the fact that Yunnan Program was more effective in raising public health literacy in indigenous populations than the National Program only. While numerous studies have shown that health literacy can be enhanced through education or communication initiatives, there is relatively little research on interventions which evaluated program targeting populations in Southwestern China and how such interventions may be optimized with culturally sensitive services (20, 41). Findings from several systematic reviews indicated that differences in the health education communication constructs employed by an intervention program, such as types of content, media, communicators and receivers, affected health education outcomes (20, 29). Building upon the National Program, a set of culturally sensitive health education components was added to the Yunnan Program by the Yunnan Provincial Health Commission to address the low health literacy of residents in two severely impoverished prefectures. This additional protocol in the Yunnan Program aimed to develop a practical and feasible implementation model to accelerate the improvement of health literacy in local populations.

The design of Yunnan program followed the three well-defined guidelines for creating social epidemics described by Malcolm Gladwell (42). First, the Law of the Few states that the social epidemic is often initiated by a few motivated persons, such as mavens, connectors (i.e., those who can diffuse ideas through extensive social networks) and salesmen. As mentioned above, an important difference between National Program and Yunnan Program lied in communicator selection. While the health education team was composed mainly of trained village leaders, and primary health workers in the National Program, the delivery of Yunnan program was led by health specialists from prefecture and county-levels who played an important role in the success of Yunnan Program. The effective implementation of health education depends on both knowledge dissemination and the practice of effective interpersonal communication. In rural areas, health workers at the village level may have difficulty accessing updated information. With the increase of basic medical care and public health services in rural China (43), it is difficult to carry out meaningful health education relying solely on village doctors. Even if essential training is provided to these personnel, the practical ability to carry out health education would not be developed in the short term. Currently, most health literacy interventions are developed and led by experienced physicians (i.e., mavens) in clinical settings rather than in population-based campaigns (20). Such professionally-designed programs often realize their intent to improve the health literacy of the target audience. In our study, the Yunnan Program also adopted this approach. By establishing a specialized team consisting of health specialists from prefecture- and county-levels, health education lectures were consistently carried out across the county. This specialized education team acted as mavens driving the spread of health knowledge as expected by the design of the Yunnan Program. Moreover, in addition to residents, the leading cadres, officials, teachers and medical doctors were also recruited as participants in the Yunnan Program. Due to their relatively high education background, these participants were more receptive to health knowledge. Additionally, the increased centrality in their social networks allowed them to play the role of “connectors” to facilitate the disseminating information in populations more widely and quickly. Thus, the employment of a professionally-developed program and the inclusion of motivated, well-connected individuals in the target group facilitated the rapid spread of educational material in the Yunnan Program.

The second prominent rule described by Gladwell is the “stickiness” of media applied by education programs, which describes the acceptability and understandability of messages among different audiences. Previous studies indicated a range of useful approaches which helped improve health education outcomes among populations with lower literacy levels, including adding video or oral communication (44, 45) or using less written narratives (46), providing culturally-appropriate health education materials (47) and using an integrated language learning curriculum for non-native language speakers (48). Our study identified education level as the most important factor affecting the level of health literacy. Most of the poor in our study sites had lower education levels, and many residents were ethnic minorities with different languages and cultures. In order to effectively spread health knowledge among such groups, the Yunnan Program employed radio dramas and advertisements with different versions of minority languages. These non-verbal and culturally-sensitive media focusing on promoting lifestyle modification were developed explicitly for low-literacy and low-acculturated populations to increase the “stickiness” of information, making health knowledge memorable and compelling.

The third rule addresses the social environment in which information and new ideas are transmitted. In the area of health promotion, settings-based approaches are often used to create supportive social environment. This type of approach was derived from an ecological perspective and “whole system thinking” that integrated health promotion concepts into the local cultural contexts and the routine living and working environments of the target audience (49). With the main purpose of developing broader corporate social responsibility, this healthy settings approach highlighted an empowerment strategy for health promotion (50). This settings-based approach offers an effective way to enhance the impact of health promotion projects through fostering leadership and advocacy in wider organizations (51). Under the Yunnan program, various institutions including hospitals, schools, government sectors and public spaces, were encouraged to create health-promoting settings in the project counties. The Yunnan Program focused on creating an inclusive and supportive environment where health can be defined, understood and promoted, going beyond of targeting individual skills and behaviors (52).

In terms of specific dimensions of health literacy, this study showed positive impacts of the combined interventions of the National and Yunnan Program particularly for chronic disease prevention and self-management in relation to a health-promoting lifestyle. These results are very encouraging but not surprising, given the fact that the added content for the Yunnan Program focused on lifestyle-oriented learning. At present, health education programs developed in many countries have shown good results, but most programs target highly specific topics, such as comprehending food labels (53) or promoting self-care among diabetic or cardiac patients (45). Compared with the National Program, the Yunnan Program prioritized healthy lifestyle promotion. As a result, after implementing the project, the improvement of health literacy in the dimensions of chronic diseases and healthy lifestyle was the highest, while the improvement of other health-related aspects was relatively modest, and health literacy on infectious diseases and health information were not equally improved. The results implied that a more targeted approach toward these aspects of health literacy may enhance the retention of this information.

As a final note, this study has three main limitations. Firstly, we employed a quasi-experiential design in this real-world implementation research to gauge the effectiveness of our program services by comparing the differences in key health literacy outcomes over time and between groups. While we have adjusted a range of confounding factors in the regression models, there could be potential confounders that were not captured by the data we collected. The second limitation involved a lack of measurement to monitor the level and pattern of program engagement at the participant and community levels. We were unable to examine whether and how program engagement affected health literacy outcomes in the target population. It is emphasized by WHO and experts from the field that the empowerment of individuals and community in health promotion is critical for the success of health education program (41, 54, 55). We will incorporate the collection of engagement indicators in our future program to examine the effect of program engagement on health literacy outcomes. Another limitation of this study lied in that we were not able to collect survey data from individuals who were under the age of 15 or over the age of 70 due to the restrictions on visits to schools and the senior citizens living in the community during the COVID-19 pandemic. Findings from this analysis did not capture the effect of our healthy literacy intervention on these two subgroups of the population who were also exposed to program intervention at the program sites.

## Data availability statement

The original contributions presented in the study are included in the article/supplementary material, further inquiries can be directed to dengruirita@126.com.

## Author contributions

RD and YH conceptualized and designed the study. BL and YH conducted the statistical analyses. BL, RD, YH, and FJ drafted the initial manuscript. HF and CL edited and revised the manuscript. All authors interpreted the results and reviewed and approved the final manuscript as submitted.
